# Supplementary material for: Antigen discrimination by T cells relies on size-constrained microvillar contact
Source: Nat Commun. 2023 Mar 23;14:1611. doi: 10.1038/s41467-023-36855-9 (PMC10036606; doi:10.1038/s41467-023-36855-9)
Supplement: Supplementary file 1 — Supplementary Information [file 41467_2023_36855_MOESM1_ESM.pdf]

Supplementary Information

Antigen discrimination by T cells relies on size-constrained microvillar contact

Edward Jenkins, Markus Körbel, Caitlin O'Brien-Ball, James McColl, Kevin Y. Chen, Mateusz Kotowski, Jane Humphrey, Anna H. Lippert, Heather Brouwer, Ana Mafalda Santos, Steven F. Lee, Simon J. Davis, David Klenerman

Inventory

1) Supplementary figures and legends 1-6

Supplementary Figures

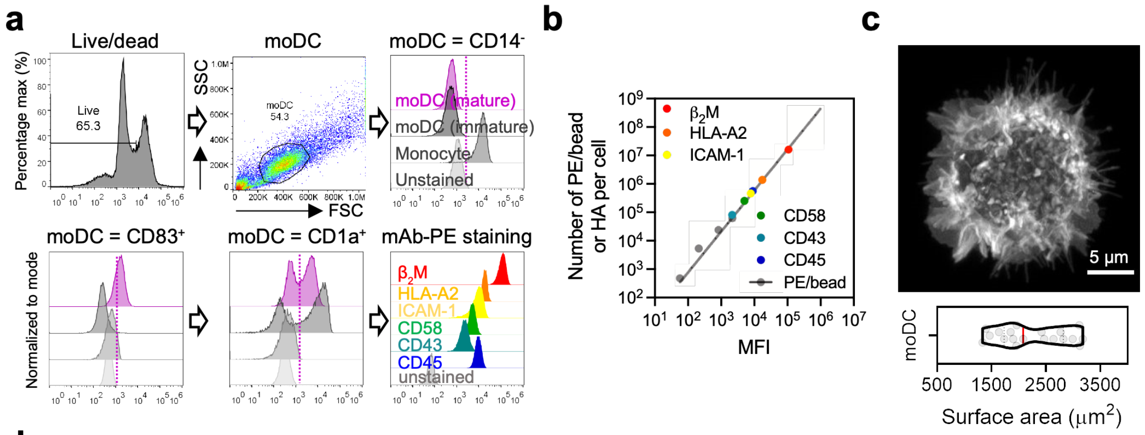

d

Protein	Density of molecules (µm^{-2})
β_2M	6450-10,700
HLA-A2	660-870
ICAM-1	265-445
CD58	110-170
CD43	40-60
CD45	270-450

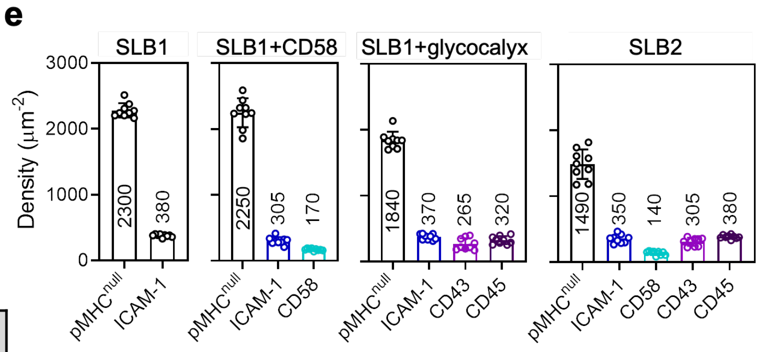

f

Protein (cell type)	Total protein count (median)
CD2 (J8-GECI)	40,700
CD2 (primary CD8⁺)	29,750
LFA-1 (J8-GECI)	46,320
LFA-1 (primary CD8⁺)	50,500
CD43 (J8-GECI)	2,700
CD43 (primary CD8⁺)	1,060
CD45 (J8-GECI)	106,300
CD45 (primary CD8⁺)	129,850

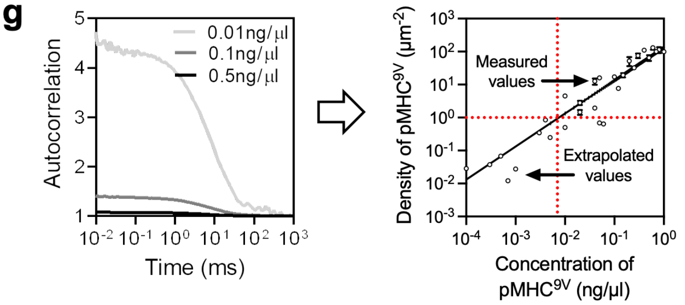

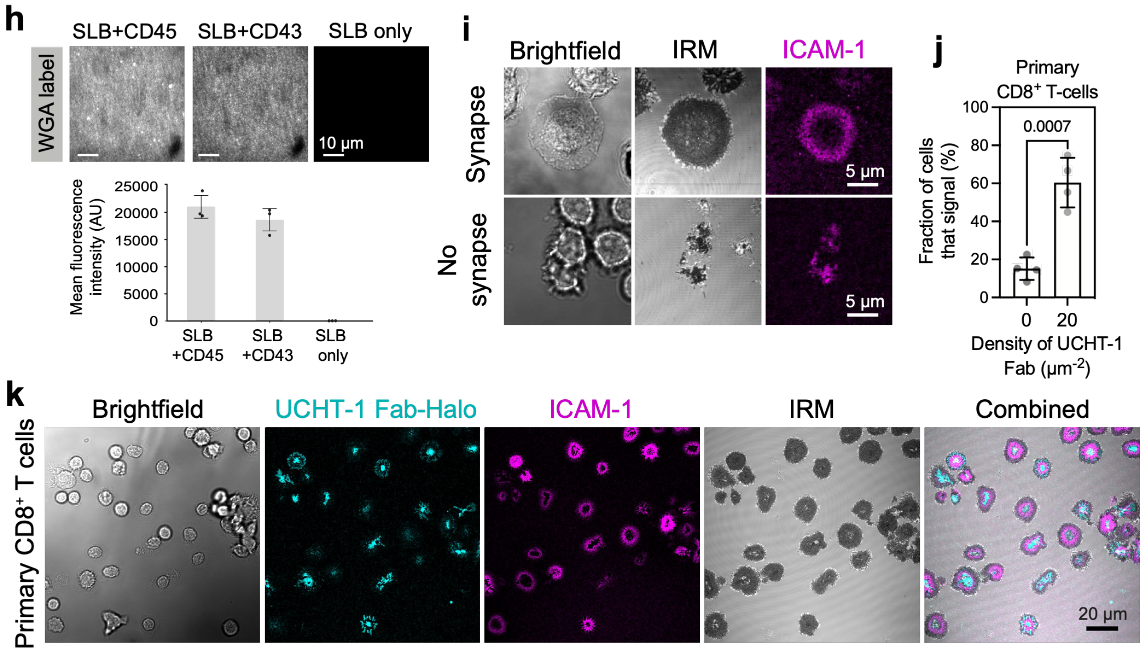

Supplementary Figure 1. Creation of SLB2s

a Gating strategy for mature monocyte-derived dendritic cells (moDCs). **b** Quantibrite analysis of key surface proteins on moDCs. Each point is the geometric mean of the histograms taken in **a**. MFI = median fluorescence intensity. **c** Airyscan max z-projection of a fixed moDC labeled with membrane dye. Quantification of the total surface area is shown below (n = 19 cells; see Methods). Red line is the median surface area. **d** Density (± 1 x S.D.) of key surface proteins on moDCs. Quantibrite measurements in **b** were divided by the median surface area in **c**. **e** Density measurements of proteins on SLBs. The densities of each protein on the SLBs were matched to those on moDCs (see **d**), measured using point fluorescence correlation spectroscopy (pFCS). Each bar is the mean (± S.D.) of nine 10s measurements from three SLBs. **f** Quantibrite analysis of key surface proteins on J8-GECI or primary $CD8^+$ T-cells. Data representative of three independent repeats. **g** Analysis pipeline for altering and measuring $pMHC^{9V}$ density. To titrate $pMHC^{9V}$ in a linear manner, while keeping total protein constant on the SLB, $pMHC^{null}$ was exchanged at a molar ratio for $pMHC^{9V}$. Left plot shows example pFCS measurements for several $pMHC^{9V}$ concentrations. Density values below ~1 molecule/μm^2 were extrapolated from a four-point linear regression with the curve forced to pass through (0,0). Right plot shows relationship between $pMHC^{9V}$ concentration and density for SLB1 (n = 82 independent SLB values with the mean ± S.D. shown for repeat concentrations measured). The same was done for other SLB compositions (see Methods for equation values). The slope of the fitted line was used to set $pMHC^{9V}$ densities in subsequent experiments. **h** Representative images showing the presence of sugar groups (*i.e.,* sialic acid) on SLBs presenting CD45 or CD43, but not in their absence. Sugar groups were labelled using fluorescently tagged wheat germ agglutinin (WGA). Bar graph shows the mean (± S.D) intensity value of tagged WGA on an SLB for n = 3 SLBs. **i** Representative example and digital zoom taken from a repeat in Fig. 1e of a J8-GECI cell forming a 'synapse', *i.e.,* a large dark zone in IRM and a ring of ICAM-1 accumulation, or 'no synapse', *i.e.,* a small dark zone in IRM and irregular ICAM-1 accumulation. Cells with no ICAM-1 accumulation were categorized as 'no synapse'. **j** Fraction of primary $CD8^+$ T-cells exhibiting calcium release on SLB2s presenting $pMHC^{null}$ ± ~20 molecules/μm^2 of UCHT-1 Fab-HaloTag. Shown is the mean (± S.D.) of n = 4 SLBs with ≥168 cells analyzed per SLB. Means were compared using a two-sided Student's t-test. **k** Representative example from n = 3 SLBs of primary $CD8^+$ T-cells forming synapses on an SLB2 presenting $pMHC^{null}$ + ~20 molecules/μm^2 of UCHT-1 Fab-HaloTag. Source data are provided in the Source Data file.

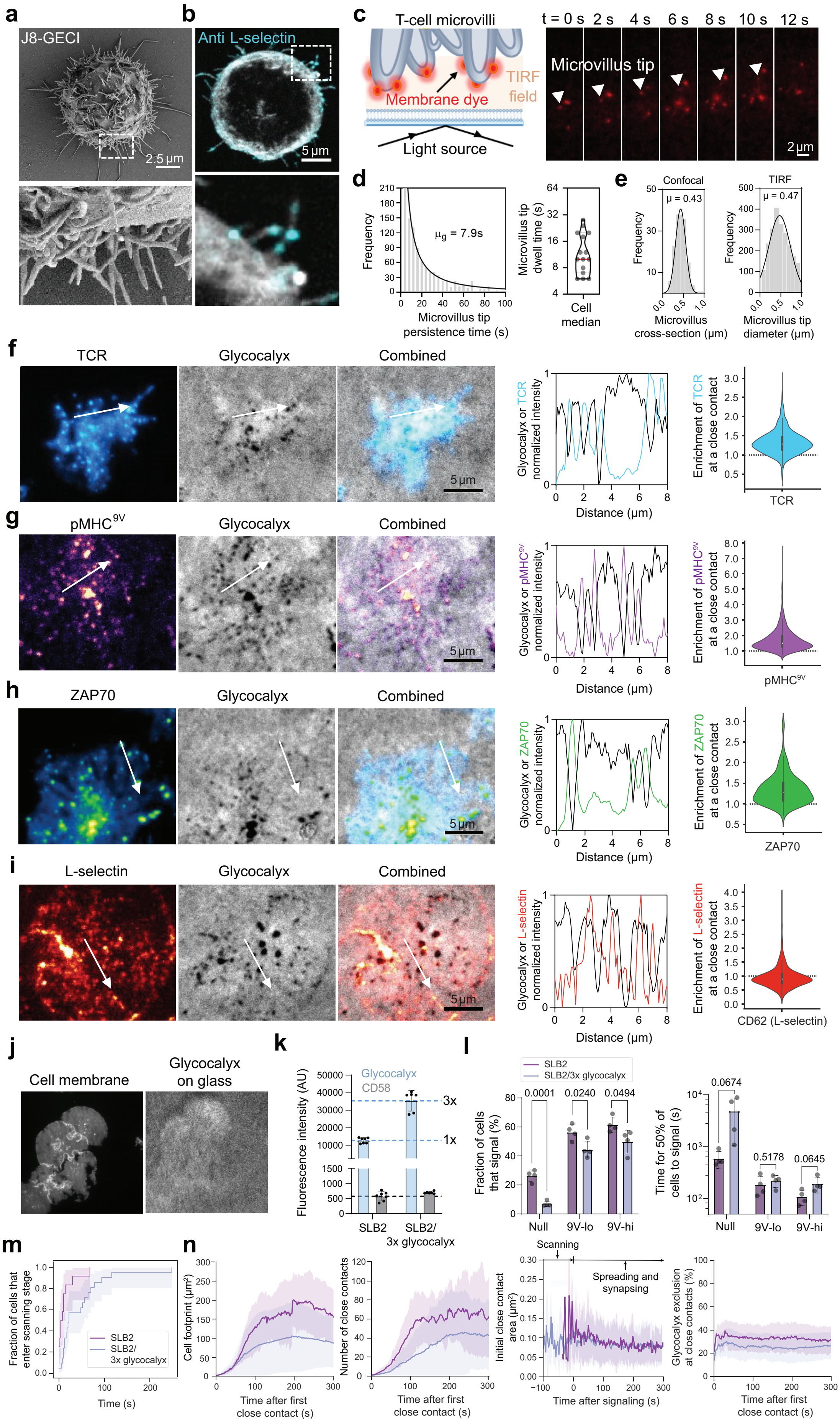
a
J8-GECI
2.5 µm
b
Anti L-selectin
5 µm
c
T-cell microvilli
Membrane dye
TIRF field
Light source
t = 0 s
2 s
4 s
6 s
8 s
10 s
12 s
Microvillus tip
2 µm
d
Frequency
μ_g = 7.9s
Microvillus tip persistence time (s)
Microvillus tip dwell time (s)
Cell median
e
Confocal
μ = 0.43
Frequency
Microvillus cross-section (μm)
TIRF
μ = 0.47
Microvillus tip diameter (μm)
f
TCR
Glycocalyx
Combined
5 µm
Glycocalyx or TCR normalized intensity
Distance (μm)
Enrichment of TCR at a close contact
g
pMHC^{9V}
Glycocalyx
Combined
5 µm
Glycocalyx or pMHC^{9V} normalized intensity
Distance (μm)
Enrichment of pMHC^{9V} at a close contact
h
ZAP70
Glycocalyx
Combined
5 µm
Glycocalyx or ZAP70 normalized intensity
Distance (μm)
Enrichment of ZAP70 at a close contact
i
L-selectin
Glycocalyx
Combined
5 µm
Glycocalyx or L-selectin normalized intensity
Distance (μm)
Enrichment of L-selectin at a close contact
CD62 (L-selectin)
j
Cell membrane
Glycocalyx on glass
k
Glycocalyx
CD58
Fluorescence intensity (AU)
3x
1x
SLB2
SLB2/ 3x glycocalyx
l
SLB2
SLB2/3x glycocalyx
Fraction of cells that signal (%)
0.0001
0.0240
0.0494
Null
9V-lo
9V-hi
Time for 50% of cells to signal (s)
0.0674
0.5178
0.0645
m
Fraction of cells that enter scanning stage
SLB2
SLB2/ 3x glycocalyx
Time (s)
n
Cell footprint (μm²)
Time after first close contact (s)
Number of close contacts
Time after first close contact (s)
Scanning
Spreading and synapsing
Initial close contact area (μm²)
Time after signaling (s)
Glycocalyx exclusion at close contacts (%)
Time after first close contact (s)

Supplementary Figure 2. 'Close contact' formation by J8-GECI cells on SLB2s

a Scanning electron microscopy image of the J8-GECI cell line. Digital zoom taken from white dashed box. The image is representative of cells across n = 3 wells. **b** Confocal fluorescence image of the mid-plane of J8-GECI cells. The figure shows enrichment of L-selectin (cyan) at membrane (grey) protrusions. L-selectin is considered a marker for microvilli[56]. Digital zoom taken from white dashed box. The image is representative of cells across n = 3 wells. **c** Cartoon representation and 12 s time-series of microvillus sampling dynamics imaged using TIRFM. Image shows J8-GECI cells on an SLB2 presenting $pMHC^{null}$. Arrow indicates one microvillar tip, which disappears at 12 s as it moves out of the TIRF evanescent field. **d** Histogram of microvillar tip persistence within TIRF evanescent field (as seen in **c**) for J8-GECI cells (left); n = 4389 tracks taken from 20 cells across 3 SLBs. The short microvillar tip persistence is indicative of the dynamic probing of microvilli. μ_g is the geometric mean of the distributions, fit using a lognormal distribution in GraphPad Prism. The median track times for each cell are shown in the violin plot (right). Red line indicates median. **e** Histograms of the diameter of microvilli measured using the full-width half-maximum from cross-sections of microvilli in confocal imaging (as seen in **b**; data pooled from n = 11 cells with 125 total microvilli analyzed) or from the diameter of probing microvillar tips (as shown in **c**; data pooled from n = 20 cells with 2294 total microvillar tips analyzed). μ is the mean of the distribution, fit with a Gaussian distribution in GraphPad Prism. **f-i** Representative TIRF images, line profiles (along and in direction of white arrow) and enrichment plots of key proteins in relation to close contacts (*i.e.,* holes in the glycocalyx channel) formed by microvilli breaching the SLB glycocalyx. Images show J8-GECI cells on an SLB2 presenting $pMHC^{null}$+$pMHC^{9V\text{-}hi}$ (*i.e.,* 100 molecules $pMHC^{9V}/\mu m^2$); n = 843 (**f**, 17 FOVs), 1219 (**g**, 8 FOVs), 201 (**h,** 2 FOVs), and 1068 (**i**, 4 FOVs) total contacts analyzed to produce the enrichment plots. Values below and above 1 indicate exclusion and enrichment, respectively. The boxplots indicate the quartiles with a line at the median. Whiskers extend to points that lie within 1.5 IQRs of the lower and upper quartile. Close contact formation relative to **f** TCR (see related Supplementary Movie 13), **g** $pMHC^{9V}$ (see related Supplementary Movie 8), **h** ZAP70 (see related Supplementary Movie 9), and **i** L-selectin (see related Supplementary Movie 2). **j** Image of J8-GECI cells interacting with a glass slide coated with SLB2 proteins. Holes in glycocalyx fluorescence do not form at areas of cell membrane fluorescence. The image is representative of cells across n = 2 SLBs. **k** Fluorescence intensity of either CD58 or the glycocalyx (CD45 and CD43) on SLB2s or SLB2s with a 3-fold denser glycocalyx (SLB2/3X glycocalyx). Each bar is the mean intensity (± S.D.) of n = 7 (SLB2) and 6 (SLB2/3X glycocalyx) SLBs. **l** Fraction of J8-GECI cells exhibiting calcium release (left plot) or time for 50% of all cells to show calcium release (right plot) on an SLB2 or SLB2/3X glycocalyx presenting $pMHC^{null}$ ± 1 (9V-lo) or 100 molecules (9V-hi)/μm^2 of $pMHC^{9V}$. Shown is the mean (± S.D.) of n = 4 SLBs with ≥80 cells analyzed per SLB. Means were compared using a two-sided Student's t-test. **m** Image analysis of J8-GECI cells on SLB2 or SLB2/3X glycocalyx surfaces presenting $pMHC^{null}$+$pMHC^{9V\text{-}hi}$; n = 12 (SLB2, 2 SLBs), and 21 (SLB2/3X glycocalyx, 3 SLBs) cells. The cumulative distribution of the searching to scanning stage transition is shown. See Methods for more details of analysis. **n** Image analysis of signaling cells. (First plot) Cell footprint over time, plotted relative to the first appearance of a close contact (timepoint t = 0 s). Plotted is the mean (± S.D.). (Second plot) Number of close contacts over time. Plotted is the mean (± S.D.). (Third plot) Area of individual close contacts within the first 10 s after their formation. Plotted is the mean (± S.D.). (Fourth plot) Glycocalyx exclusion at individual close contacts pooled from all cells over time. Plotted is the mean (± S.D.). n(cells) = 4 (SLB2) and 16 (SLB2/3X glycocalyx) from the same SLBs as in **m**. Source data are provided in the Source Data file.

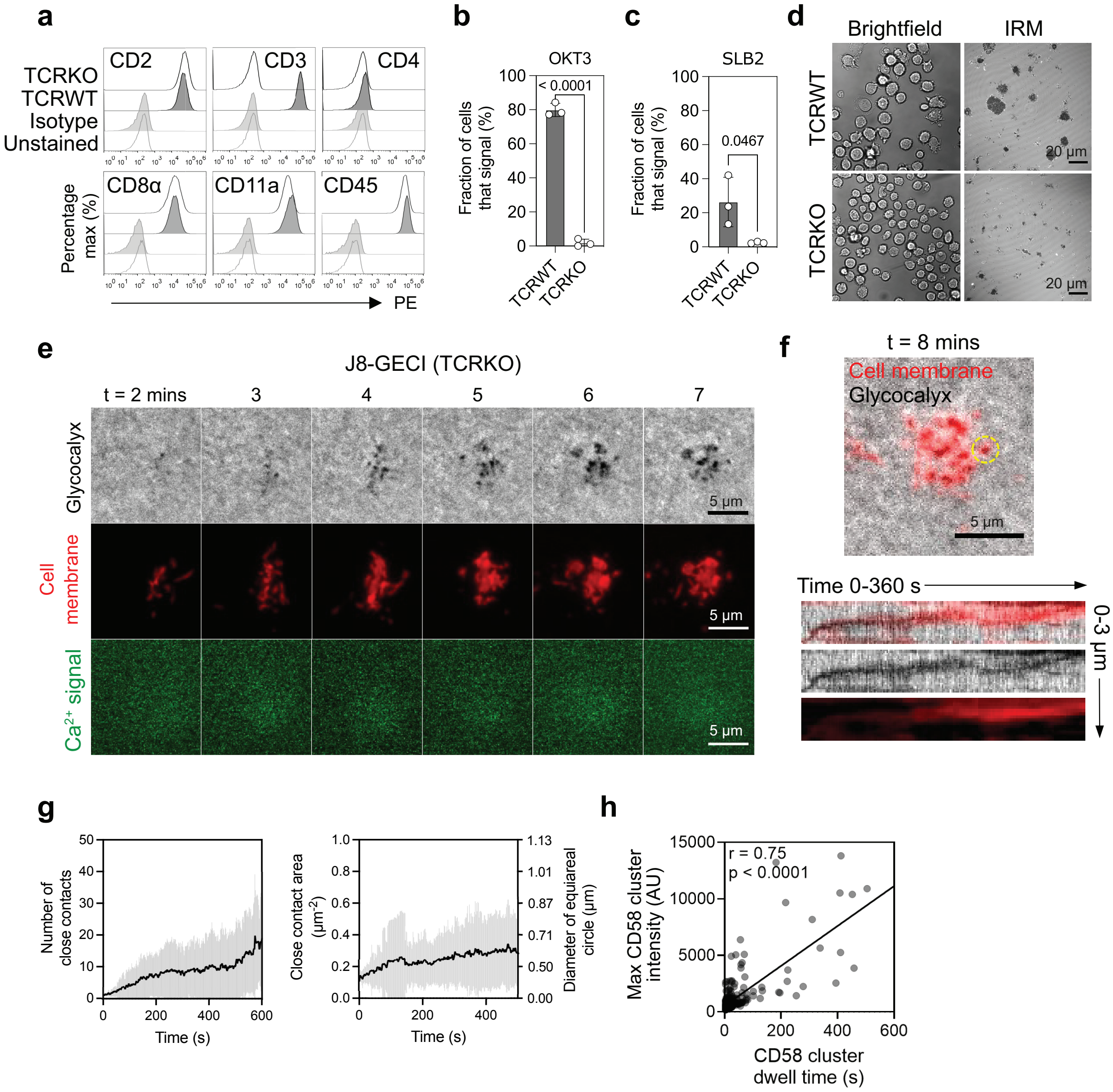
a
TCRKO
TCRWT
Isotype
Unstained
CD2
CD3
CD4
CD8α
CD11a
CD45
Percentage max (%)
PE
b
OKT3
Fraction of cells that signal (%)
< 0.0001
TCRWT
TCRKO
c
SLB2
Fraction of cells that signal (%)
0.0467
TCRWT
TCRKO
d
Brightfield
IRM
TCRWT
TCRKO
20 μm
e
J8-GECI (TCRKO)
t = 2 mins
3
4
5
6
7
Glycocalyx
Cell membrane
Ca2+ signal
5 μm
f
t = 8 mins
Cell membrane
Glycocalyx
5 μm
Time 0-360 s
0-3 μm
g
Number of close contacts
Time (s)
Close contact area (μm2)
Diameter of equiareal circle (μm)
Time (s)
h
r = 0.75
p < 0.0001
Max CD58 cluster intensity (AU)
CD58 cluster dwell time (s)

Supplementary Figure 3. Calcium release and spreading depend on the TCR, but close contact formation and stabilization are independent of the TCR

a Flow cytometry histograms of key surface proteins on J8-GECI (TCRWT) and TCR-deficient J8-GECI (TCRKO) cells. **b** Calcium release on OKT3-coated glass. Shown is the mean (± S.D.) of n = 3 wells with ≥233 cells analyzed per well. Means were compared using the two-sided Student's t-test. **c** Calcium release on an SLB2 presenting pMHC^{null}. Shown is the mean (± S.D.) of n = 3 SLBs with ≥481 cells analyzed per SLB. Means were compared using the two-sided Student's t-test. **d** Representative brightfield and IRM images from n = 3 SLBs taken immediately after the 10-minute calcium tracking movies (data shown in **c**). **e** Representative images of microvillar close contact formation with TCRKO cells on an SLB2 presenting pMHC^{null}. See Supplementary Movie 11. **f** Close contact formation and kymograph of a single stabilized close contact (indicated by yellow-dashed circle) tracked over 360 s. **g** Analysis of close contact formation for TCRKO cells on an SLB2 presenting pMHC^{null}. Shown are the number of close contacts formed (left) and close contact area (or diameter of equiareal circle, right) versus time. Data shows mean (± S.D.) of contacts taken from n = 16 cells over 3 SLBs. **h** CD58 cluster dwell time (a proxy for close contact dwell time) correlates with cluster intensity (a proxy for total CD58 at a close contact); n = 225 tracks from 5 cells. Data was analyzed using a two-sided Pearson correlation analysis. Source data are provided in the Source Data file.

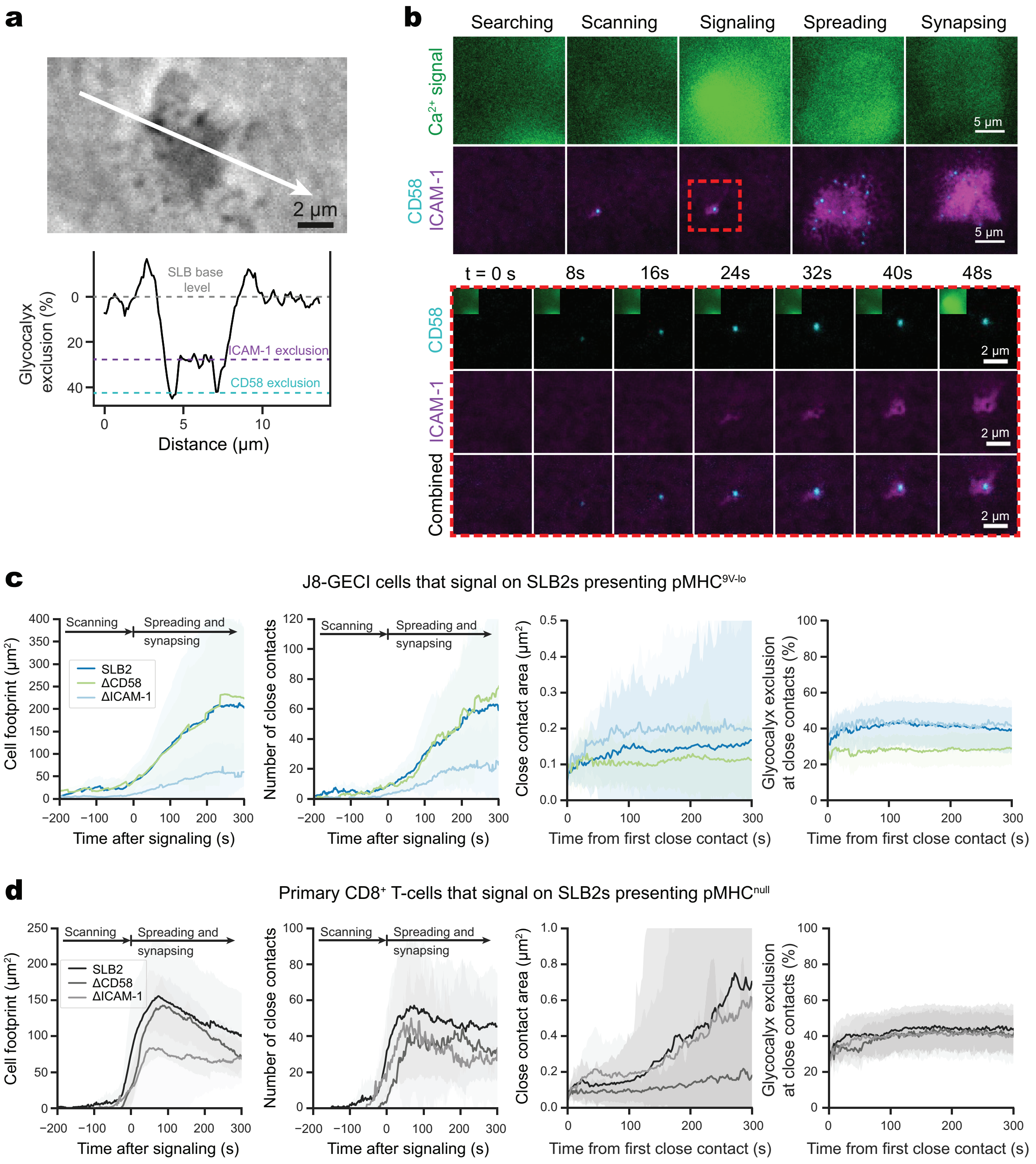

a
2 μm
SLB base level
ICAM-1 exclusion
CD58 exclusion
Glycocalyx exclusion (%)
Distance (μm)
b
Searching
Scanning
Signaling
Spreading
Synapsing
Ca2+ signal
CD58
ICAM-1
5 μm
t = 0 s
8s
16s
24s
32s
40s
48s
Combined
2 μm
c
J8-GECI cells that signal on SLB2s presenting pMHC9V-lo
Scanning
Spreading and synapsing
SLB2
ΔCD58
ΔICAM-1
Cell footprint (μm2)
Number of close contacts
Close contact area (μm2)
Glycocalyx exclusion at close contacts (%)
Time after signaling (s)
Time from first close contact (s)
d
Primary CD8+ T-cells that signal on SLB2s presenting pMHCnull
Scanning
Spreading and synapsing
SLB2
ΔCD58
ΔICAM-1
Cell footprint (μm2)
Number of close contacts
Close contact area (μm2)
Glycocalyx exclusion at close contacts (%)
Time after signaling (s)
Time from first close contact (s)

Supplementary Figure 4. Impact of CD2 and LFA-1 across four stages of contact

a Exclusion of the glycocalyx from areas of CD58 (black spots) and ICAM-1 (darker grey areas) accumulation (top). The intensity line profile was taken along and in the direction of the white arrow (bottom). Exclusion is the fractional (%) decrease in the SLB glycocalyx fluorescence from the baseline. **b** CD58 and ICAM-1 accumulation at different stages of close contact formation. Shown is a J8-GECI cell on an SLB2 presenting $pMHC^{null}$+$pMHC^{9V\text{-}lo}$. Bottom three rows are a digital zoom (indicated by dashed red box) of a single 'micro adhesion ring' forming prior to calcium release. The images are representative of J8-GECI cells taken from n = 3 SLBs. **c** Quantitative image analysis of J8-GECI cells on SLB2, SLB2ΔCD58, or SLB2ΔICAM-1 surfaces, all presenting $pMHC^{9V\text{-}lo}$. (First plot) Cell footprint (cell membrane area) versus time relative to the timepoint of TCR triggering (*i.e.,* calcium release). Plotted is mean (± S.D.) from n = 17 (SLB2, from 5 SLBs), 11 (ΔCD58, from 3 SLBs), and 7 (ΔICAM-1, from 2 SLBs) cells. (Second plot) Number of close contacts formed versus time relative to timepoint of calcium release. Plotted is mean (± S.D.). (Third plot) Size of individual close contacts from all cells versus time. Plotted is mean (± S.D.). (Fourth plot) Glycocalyx exclusion at individual close contacts from all cells versus time. **d** Same as **c** but for primary $CD8^+$ T-cells that exhibit calcium release on SLB2s, or SLB2ΔCD58 or SLB2ΔICAM-1 surfaces, all presenting $pMHC^{null}$; n = 14 (SLB2, from 6 SLBs), 2 (ΔCD58, from 3 SLBs), and 3 (ΔICAM-1, from 2 SLBs) cells. Experiments are the same as in Fig. 4f-h. Source data are provided in the Source Data file.

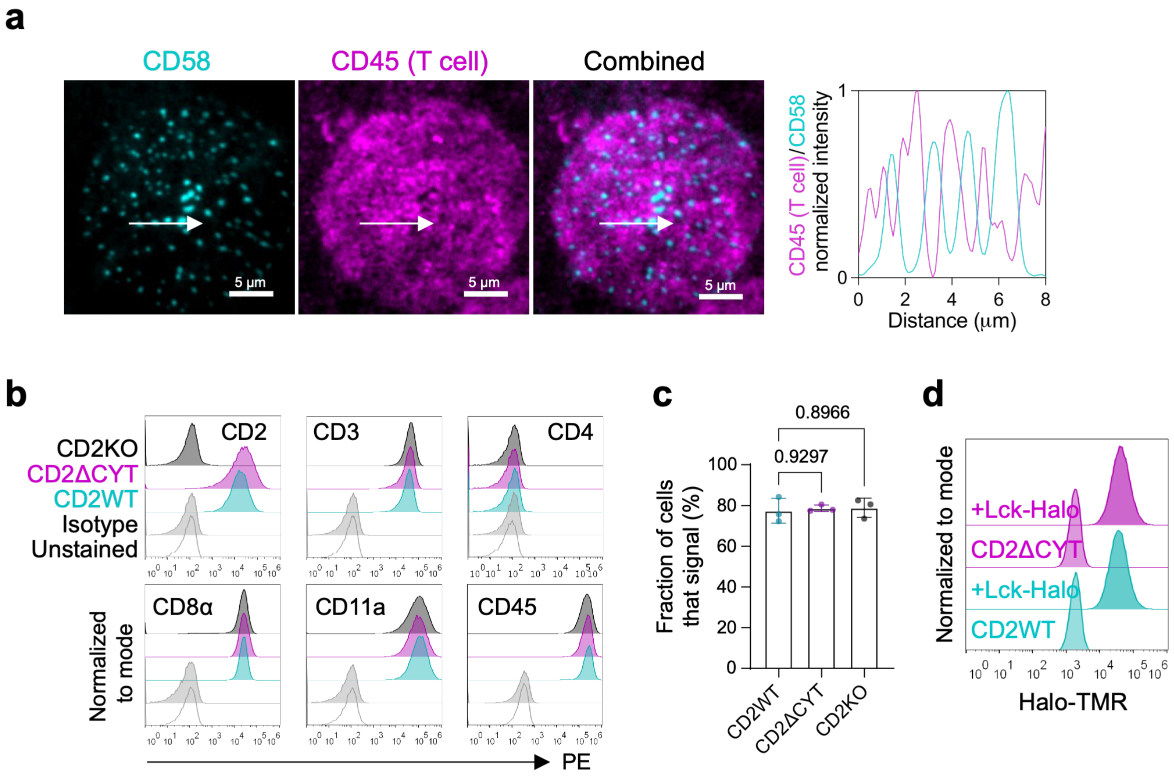

Supplementary Figure 5. CD45 exclusion and cell line generation

a Exclusion of CD45 (labeled with anti-CD45 antibody, clone Gap8.3, magenta) from areas of CD58 (cyan) accumulation for a J8-GECI cell at the 'spreading' stage of close contact formation on an SLB2 presenting pMHC^{null}+pMHC$^{9V\text{-}lo}$ (left). The min/max normalized intensity line profile was taken along and in the direction of the white arrow (right). The image is representative of J8-GECI cells taken from n = 3 SLBs. **b** Flow cytometry histograms of key surface receptors. **c** Fraction of cells that exhibit calcium release on OKT3-coated glass. Shown is the mean (± S.D.) of n = 3 coated wells with ≥190 cells analyzed per well. Means were compared using one-way ANOVA with Dunnett's correction with CD2WT as the control group. **d** Flow cytometry histograms showing comparable labeling of Lck-HaloTag using HaloTag TMR in the CD2WT and CD2ΔCYT cell lines. Source data are provided in the Source Data file.

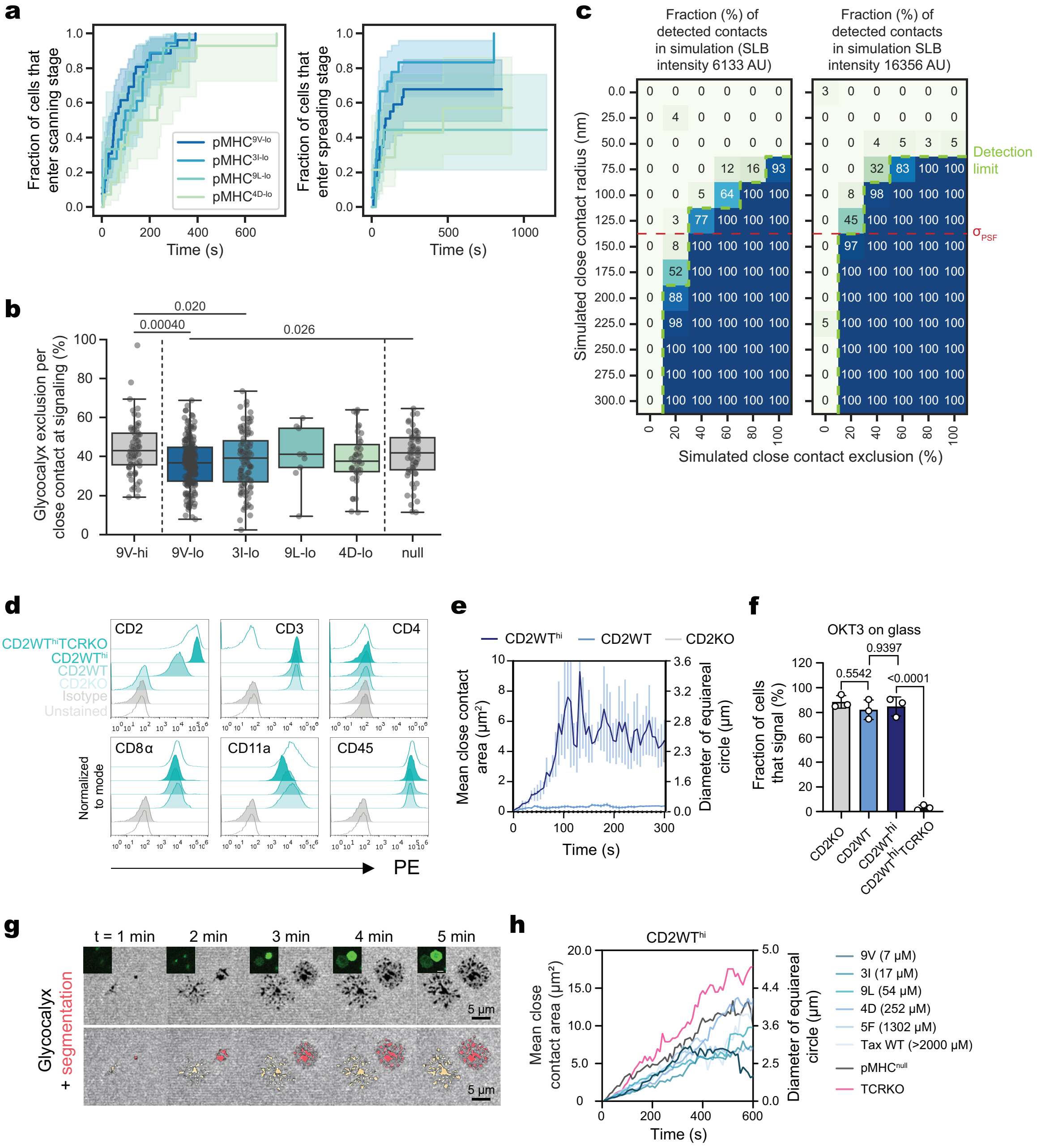
a
Fraction of cells that enter scanning stage
Time (s)
pMHC9V-lo
pMHC3I-lo
pMHC9L-lo
pMHC4D-lo
Fraction of cells that enter spreading stage
Time (s)
b
Glycocalyx exclusion per close contact at signaling (%)
0.020
0.00040
0.026
9V-hi
9V-lo
3I-lo
9L-lo
4D-lo
null
c
Fraction (%) of detected contacts in simulation (SLB intensity 6133 AU)
Fraction (%) of detected contacts in simulation SLB intensity 16356 AU)
Simulated close contact radius (nm)
Simulated close contact exclusion (%)
Detection limit
σPSF
d
CD2WThiTCRKO
CD2WThi
CD2WT
CD2KO
Isotype
Unstained
CD2
CD3
CD4
CD8α
CD11a
CD45
Normalized to mode
PE
e
CD2WThi
CD2WT
CD2KO
Mean close contact area (μm²)
Diameter of equiareal circle (μm)
Time (s)
f
OKT3 on glass
Fraction of cells that signal (%)
0.9397
0.5542
<0.0001
CD2KO
CD2WT
CD2WThi
CD2WThiTCRKO
g
t = 1 min
2 min
3 min
4 min
5 min
Glycocalyx + segmentation
5 μm
5 μm
h
CD2WThi
Mean close contact area (μm²)
Diameter of equiareal circle (μm)
Time (s)
9V (7 μM)
3I (17 μM)
9L (54 μM)
4D (252 μM)
5F (1302 μM)
Tax WT (>2000 μM)
pMHCnull
TCRKO

Supplementary Figure 6. Measuring and altering close contacts

a Cumulative distribution of the searching to scanning stage (left) and scanning to spreading stage (right) transition for J8-GECI cells on SLB2s presenting $pMHC^{null}$ + 1 molecule/μm^2 of the indicated agonist pMHC. The analysis uses both signaling and non-signaling cells from the same experiment as in Fig. 6c; n = 26 ($9V^{lo}$, from 5 SLBs), 18 ($3I^{lo}$, from 4 SLBs), 12 ($9L^{lo}$, from 3 SLBs), and 14 ($4D^{lo}$, from 2 SLBs) cells. Plotted are the cumulative distribution functions of the Kaplan-Meier estimator with the exponential Greenwood confidence interval. **b** Glycocalyx exclusion at close contacts formed by J8-GECI cells interacting with SLB2s presenting $pMHC^{null}$ plus the indicated agonist pMHC affinity/density, at the time of calcium release. The boxplots indicate the quartiles with a line at the median. Whiskers extend to points that lie within 1.5 IQRs of the lower and upper quartile. Distributions were compared using the two-sided Mann-Whitney U test and p-values <0.05 are shown. **a**, **b** Data are from the same experiment shown in Fig. 6c. **c** Fraction of detected contacts for a given close contact radius and extent of exclusion of the glycocalyx. Images were simulated assuming a homogeneous SLB fluorescence of two different intensities (6133 and 16356 arbitrary units, *i.e.,* within the range of experimental data). Image formation was simulated with a Gaussian blur of σ_{PSF} = 131 nm (red dotted lines). At 40% exclusion (*i.e.*, the average experimentally measured exclusion, Fig. 4d) the analysis can detect >75% of contacts with a diameter >250 nm (worst case) and >200 nm (best case). The detection limit (green dotted lines) indicates conditions in which over 75% of contacts can be detected. Given is the number of frames a contact was detected in a simulated timelapse with 100 frames. **d** Flow cytometry histograms showing comparable expression of key surface receptors. **e** Mean close contact area versus time for the indicated cell lines on SLB2s presenting $pMHC^{null}$. Data show the mean (± S.D.) of the contact area from n = 11 (CD2KO), 9 (CD2WT), and 3 ($CD2WT^{hi}$) cells. Time point 0 s corresponds to the time that the first contact was detected. **f** Fraction (%) of cells that exhibit calcium release on OKT3-coated glass. Shown is the mean (± S.D.) of n = 3 wells with ≥150 cells analyzed per SLB. One-way ANOVA with Šídák's correction was used to compare means. **g** Confocal fluorescence images and segmentation of close contacts formed by $CD2WT^{hi}$ cells on SLB2s presenting $pMHC^{null}$. See related Supplementary Movie 24**.** The image is representative of cells taken from n = 3 SLBs. **h** Mean close contact area versus time for $CD2WT^{hi}$ cells on SLB2s presenting $pMHC^{null}$ ± ~10 molecules/μm^2 of agonist pMHC of varying affinities. Each line represents the mean contact area of n = 76 (9V), 75 (3I), 108 (9L), 87 (4D), 84 (5F), 91 (Tax WT), 69 ($pMHC^{null}$), and 74 (TCRKO) cells taken from three independent SLBs. Source data are provided in the Source Data file.
