# Supplementary material for: Antigen discrimination by T cells relies on size-constrained microvillar contact
Source: Nat Commun. 2023 Mar 23;14:1611. doi: 10.1038/s41467-023-36855-9 (PMC10036606; doi:10.1038/s41467-023-36855-9)
Supplement: Supplementary file 3 — Description of Additional Supplementary Files [file 41467_2023_36855_MOESM3_ESM.pdf]

## Description of Additional Supplementary Files

**Supplementary Movie 1**
**Stages of close-contact formation**
A representative three-colour TIRF movie of a J8-GECI cell showing the four stages of microvillar-based close-contact formation: ‘searching’, ‘scanning’, ‘spreading’, and ‘synapsing’ (see text for details). The movie shows a cell labelled with CellMask Deep Red interacting with an SLB2 presenting $pMHC^{null}$ plus $pMHC^{9V\text{-}hi}$. The GECI used to visualise calcium release was based on circularly permutated EGFP. The SLB glycocalyx (i.e., CD43 and CD45) used to observe close contacts was labelled with Alexa-555, and the cell membrane with CellMask Deep Red. Scale bar is 5 µm. (MP4 80506 kb.)

**Supplementary Movie 2**
**L-selectin at close contacts**
A representative three-colour TIRF movie showing that L-selectin on J8-GECI cells is present at the first appearance of microvillar close contacts (i.e., the appearance of black holes in the SLB glycocalyx fluorescence) but is rapidly excluded upon their stabilisation. The movie is of a J8-GECI cell interacting with an SLB2 presenting $pMHC^{null}$ plus $pMHC^{9V\text{-}hi}$, which induces signaling in most cells. The SLB glycocalyx was labelled with Alexa-555, and L-selectin was detected with Alexa-647 labelled anti L-selectin antibody. Scale bar is 5 µm. (MP4 23873 kb.)

**Supplementary Movie 3**
**Actin-modifying drug-treated cells on SLB2s**
A representative three-colour confocal movie showing the effect of actin-modifying drugs on close-contact formation. J8-GECI cells were treated for one hour with either DMSO or the actin-modifying drugs jasplakinolide (Jasplak), cytochalasin D (Cyto D) or latrunculin B (Lat B) prior to being placed onto SLB2s presenting $pMHC^{null}$ plus $pMHC^{9V\text{-}lo}$. $pMHC^{9V\text{-}lo}$ was used to mimic physiological scanning for rare antigens. The SLB glycocalyx was labelled with Alexa-555, and the cell membrane with CellMask Deep Red. Scale bar is 50 µm. (MP4 126220 kb.)

**Supplementary Movie 4**
**A J8-GECI cell on an SLB2 presenting $pMHC^{null}$ (signaling cell)**
A representative three-colour TIRF movie of a J8-GECI cell signaling while interacting with an SLB2 presenting $pMHC^{null}$. Unlike cells responding to $pMHC^{9V}$ (see Supplementary Movie 1), cells only exhibit a modest increase in cell footprint (i.e., cell membrane fluorescence) and contact number after signaling. The SLB glycocalyx was labelled with Alexa-555, and the cell membrane with CellMask Deep Red. Scale bar is 5 µm. (MP4 32250 kb.)

**Supplementary Movie 5**
**A J8-GECI cell on an SLB2 presenting $pMHC^{null}$ (non-signaling cell)**
A representative three-colour TIRF movie of a J8-GECI cell trapped at the scanning stage (i.e., exhibiting no calcium release), while interacting with an SLB2 presenting $pMHC^{null}$. The SLB glycocalyx was labelled with Alexa-555, and the cell membrane with CellMask Deep Red. Scale bar is 5 µm. (MP4 25989 kb.)

**Supplementary Movie 6**
**A human primary $CD8^+$ T-cell on an SLB2 presenting $pMHC^{null}$ plus $pMHC^{9V}$**
Representative three-colour TIRF movie of a human primary $CD8^+$ T-cell exhibiting all four stages of microvillar-based close-contact formation. Cells were labelled with CellMask Deep Red and Fluo-4 before being placed onto an SLB2 presenting $pMHC^{null}$ and 20 molecules/µm$^2$ of UCHT-1 Fab/HaloTag. Primary cells exhibit the same stages of close-contact formation observed for J8-GECI cells in response to $pMHC^{9V}$ (see Supplementary Movie 1), albeit with slightly different kinetics and contact parameters (see main text). Calcium release was visualised using Fluo-4 dye. The SLB glycocalyx was labelled with Alexa-555, and the cell membrane with CellMask Deep Red. Scale bar is 5 µm. (MP4 25989 kb.)

**Supplementary Movie 7**
**A human primary CD8$^{+}$ T-cell on an SLB2 presenting pMHC$^{null}$ (non-signaling cell)**
A representative three-colour TIRF movie of a primary CD8$^{+}$ T-cell trapped at the scanning stage while interacting with an SLB2 presenting pMHC$^{null}$, as observed for J8-GECI cells (see Supplementary Movie 5). pMHC$^{null}$ was used to ensure that most cells remained in the scanning stage of close-contact formation. Calcium release was visualised using Fluo-4 dye. The SLB glycocalyx was labelled with Alexa-555, and the cell membrane with CellMask Deep Red. Scale bar is 5 µm. (MP4 25989 kb.)

**Supplementary Movie 8**
**pMHC$^{9V}$ accumulation at microvillar contacts**
A representative three-colour TIRF movie of a J8-GECI cell interacting with an SLB2 presenting pMHC$^{null}$ plus pMHC$^{9V\text{-}hi}$. pMHC$^{9V}$ accumulates, and tracks over time, exclusively at microvillar close contacts. Note that, for this cell, a single contact is formed at the point of calcium release. The SLB glycocalyx was labelled with Alexa-555, and pMHC$^{9V}$ with Alexa-647. Scale bar is 5 µm. (MP4 45063 kb.)

**Supplementary Movie 9**
**ZAP70 accumulation at microvillar contacts**
A representative three-colour TIRF movie of a J8-GECI cell expressing ZAP70-Halo interacting with an SLB2 presenting pMHC$^{null}$ plus pMHC$^{9V\text{-}hi}$. There is an increase in ZAP70-Halo fluorescence/membrane recruitment at microvillar close contacts. Spots of ZAP70 accumulation also track over time with close contacts. The SLB glycocalyx was labelled with Alexa-555, and ZAP70-Halo with Janelia Fluor 646 HaloTag Ligand. Scale bar is 5 µm. (MP4 48921 kb.)

**Supplementary Movie 10**
**Single-particle tracking of pMHC$^{9V}$ at close contacts**
A representative three-colour TIRF movie of a J8-GECI cell interacting with an SLB2 presenting pMHC$^{null}$ plus pMHC$^{9V\text{-}lo}$. Due to the low density of pMHV$^{9V}$ used on the SLB, single particles can be tracked relative to close-contact formation. In this example, a single pMHC$^{9V}$ is present in a close contact at the point of calcium release. The SLB glycocalyx was labelled with Alexa-555, and pMHC$^{9V}$ with Alexa-647. Scale bar is 5 µm. (MP4 45063 kb.)

**Supplementary Movie 11**
**Close-contact formation by a TCR-deficient J8-GECI cell**
A representative three-colour TIRF movie of a TCR-deficient J8-GECI cell interacting with an SLB2 presenting pMHC$^{null}$. The movie shows that initial close-contact formation is independent of TCR/pMHC engagement and TCR signaling. The SLB glycocalyx was labelled with Alexa-555, and the cell membrane with CellMask Deep Red. Scale bar is 5 µm. (MP4 31710 kb.)

**Supplementary Movie 12**
**CD58 accumulates at close contacts**
A representative three-colour TIRF movie of a J8-GECI cell interacting with an SLB2 presenting pMHC$^{null}$ plus pMHC$^{9V\text{-}lo}$. CD58 accumulates, and tracks over time, exclusively with microvillar close contacts. The SLB glycocalyx was labelled with Alexa-555, and CD58 with Alexa-647. Scale bar is 5 µm. (MP4 45063 kb.)

**Supplementary Movie 13**
**CD58 and TCR co-localise at close contacts**
A representative three-colour TIRF movie of a J8 cell interacting with an SLB2 presenting pMHC$^{null}$ plus pMHC$^{9V\text{-}lo}$. CD58 and the TCR accumulate and co-localise, and track together over time, at microvillar close contacts. The TCR was labelled using Alexa 488-tagged anti-CD3 Fab (UCHT-1), the SLB with Alexa-555, and CD58 with Alexa-647. Scale bar is 5 µm. (MP4 45063 kb.)

**Supplementary Movie 14**
**ICAM-1 is excluded from close contacts**
A representative three-colour TIRF movie of a J8-GECI cell interacting with an SLB2 presenting $pMHC^{null}$ plus $pMHC^{9V\text{-}lo}$. ICAM-1, presumably complexed with LFA-1, is excluded from microvillar close contacts. The SLB glycocalyx was labelled with Alexa-555, and ICAM-1 with Alexa-647. Scale bar is 5 µm. (MP4 45063 kb.)

**Supplementary Movie 15**
**CD58 and ICAM-1 accumulation relative to calcium release (J8-GECI cell)**
A representative three-colour TIRF movie of a J8-GECI cell interacting with an SLB2 presenting $pMHC^{null}$ plus $pMHC^{9V\text{-}lo}$. CD58 and ICAM-1 form micro-adhesion rings (i.e., a CD58 punctum surrounded by a ring of ICAM-1). ICAM-1 was labelled with Alexa-555, and CD58 with Alexa-647. Scale bar is 5 µm. (MP4 45063 kb.)

**Supplementary Movie 16**
**CD58 and ICAM-1 accumulation relative to calcium release (primary $CD8^+$ T-cell)**
A representative three-colour TIRF movie of a human primary $CD8^+$ T-cell interacting with an SLB2 presenting $pMHC^{null}$. CD58 and ICAM-1 form micro-adhesion rings. Calcium release was visualised using Fluo-4 dye. ICAM-1 was labelled with Alexa-555, and CD58 with Alexa-647. Scale bar is 5 µm. (MP4 45063 kb.)

**Supplementary Movie 17**
**A J8-GECI cell interacting with an SLB2 lacking CD58**
A representative three-colour TIRF movie of a non-signaling cell interacting with a CD58-lacking SLB2 presenting $pMHC^{null}$. In this setting most cells remain in the scanning stage of close-contact formation. The SLB glycocalyx was labelled with Alexa-555, and the cell membrane with CellMask Deep Red. Scale bar is 5 µm. (MP4 32250 kb.)

**Supplementary Movie 18**
**A human primary $CD8^+$ T-cell interacting with an SLB2 lacking CD58**
Analogous to Supplementary Movie 17 but performed with primary $CD8^+$ T-cells. Calcium release was visualised using Fluo-4 dye. The SLB glycocalyx was labelled with Alexa-555, and the cell membrane with CellMask Deep Red. Scale bar is 5 µm. (MP4 32250 kb.)

**Supplementary Movie 19**
**A J8-GECI cell interacting with an SLB2 lacking ICAM-1**
A representative three-colour TIRF movie showing a non-signaling cell interacting with an ICAM-1-lacking SLB2 presenting $pMHC^{null}$. In this setting most cells remained in the scanning stage of close-contact formation. The SLB glycocalyx was labelled with Alexa-555, and the cell membrane with CellMask Deep Red. Scale bar is 5 µm. (MP4 32250 kb.)

**Supplementary Movie 20**
**A human primary $CD8^+$ T-cell interacting with an SLB2 lacking ICAM-1**
Analogous to Supplementary Movie 19 but performed with primary $CD8^+$ T-cells. Calcium release was visualised using Fluo-4 dye. The SLB glycocalyx was labelled with Alexa-555, and the cell membrane with CellMask Deep Red. Scale bar is 5 µm. (MP4 32250 kb.)

**Supplementary Movie 21**
**Segregation of CD45 from close contacts (J8-GECI-CD2WT cell)**
A representative three-colour TIRF movie showing the exclusion, from regions of close contact of a non-signaling J8-GECI cell with an SLB2, of cell-expressed CD45. The SLB2 presented $pMHC^{null}$, ensuring that most cells remain in the scanning stage of close-contact formation. The SLB glycocalyx was labelled with Alexa-555, and CD45 with silicon rhodamine labelled anti-CD45 (Gap8.3) antibody. Scale bar is 5 µm. (MP4 23873 kb.)

**Supplementary Movie 22**
**Segregation of CD45 from close contacts (J8-GECI-CD2ΔCYT cell)**
Analogous to Supplementary Movie 21 but using the J8-GECI-CD2ΔCYT cell line, which formed slightly less 'tight' contacts and produced less CD45 segregation versus the J8-GECI-CD2WT cell line. The SLB glycocalyx was labelled with Alexa-555, and CD45 with silicon rhodamine labelled anti-CD45 (Gap8.3) antibody. Scale bar is 5 μm. (MP4 23873 kb.)

**Supplementary Movie 23**
**Segregation of CD45 from close contacts (J8-GECI-CD2KO cell)**
Analogous to Supplementary Movie 21 but using the J8-GECI-CD2KO cell line which, like J8-GECI cells on an SLB2 lacking CD58 (see Supplementary Movie 17), formed unstable and less 'tight' close contacts (indicated by glycocalyx exclusion) without CD2. The SLB glycocalyx was labelled with Alexa-555, and CD45 with silicon rhodamine labelled anti-CD45 (Gap8.3). Scale bar is 5 μm. (MP4 23873 kb.)

**Supplementary Movie 24**
**A J8-GECI-CD2WT$^{hi}$ cell forming unconstrained close contacts on an SLB2**
A representative three-colour confocal movie with brightfield and interference reflection fluorescence microscopy (IRM), of J8-GECI cells overexpressing CD2 interacting with an SLB2 presenting pMHC$^{null}$. IRM shows that CD58 accumulation corresponds to darker areas in the IRM channel, i.e., regions of contact. The SLB glycocalyx was labelled with Alexa-555, and CD58 on the SLB, indicating CD2 engagement, with Alexa-647. Scale bar is 30 μm. (MP4 49636 kb.)
