# Supplementary material for: Antigen discrimination by T cells relies on size-constrained microvillar contact
Source: Nat Commun. 2023 Mar 23;14:1611. doi: 10.1038/s41467-023-36855-9 (PMC10036606; doi:10.1038/s41467-023-36855-9)
Supplement: Supplementary file 28 — Reporting Summary [file 41467_2023_36855_MOESM28_ESM.pdf]

nature portfolio

Corresponding author(s): Simon J. Davis
David Klenerman

Last updated by author(s): Jan 30, 2023

Reporting Summary

Nature Portfolio wishes to improve the reproducibility of the work that we publish. This form provides structure for consistency and transparency in reporting. For further information on Nature Portfolio policies, see our Editorial Policies and the Editorial Policy Checklist.

Statistics

For all statistical analyses, confirm that the following items are present in the figure legend, table legend, main text, or Methods section.

n/a	Confirmed	
☐	☒	The exact sample size (*n*) for each experimental group/condition, given as a discrete number and unit of measurement
☐	☒	A statement on whether measurements were taken from distinct samples or whether the same sample was measured repeatedly
☐	☒	The statistical test(s) used AND whether they are one- or two-sided<br>*Only common tests should be described solely by name; describe more complex techniques in the Methods section.*
☒	☐	A description of all covariates tested
☐	☒	A description of any assumptions or corrections, such as tests of normality and adjustment for multiple comparisons
☐	☒	A full description of the statistical parameters including central tendency (e.g. means) or other basic estimates (e.g. regression coefficient) AND variation (e.g. standard deviation) or associated estimates of uncertainty (e.g. confidence intervals)
☐	☒	For null hypothesis testing, the test statistic (e.g. *F*, *t*, *r*) with confidence intervals, effect sizes, degrees of freedom and *P* value noted<br>*Give P values as exact values whenever suitable.*
☒	☐	For Bayesian analysis, information on the choice of priors and Markov chain Monte Carlo settings
☒	☐	For hierarchical and complex designs, identification of the appropriate level for tests and full reporting of outcomes
☐	☒	Estimates of effect sizes (e.g. Cohen's *d*, Pearson's *r*), indicating how they were calculated

Our web collection on statistics for biologists contains articles on many of the points above.

Software and code

Policy information about availability of computer code

Data collection	Flow cytometry data with Attune Nxt (ThermoFisher Scientifc)<br>Scanning electron microscopy images with JEOL-6390 Scanning Electron Microscope<br>Confocal imaging with Zeiss LSM 780 Inverted confocal (with pFCS module) and Zeiss LSM 880 with AiryScan<br>TIRF imaging using a custom build TIRF setup using Micro-Manager software (version 2.0.0) to control imaging (Open Imaging, Inc). Details of the custom TIRF microscope setup are described in the methods.
Data analysis	Images and line profiles were prepared using FIJI (version 1.53q). The tracking of cell movement and calcium release from raw confocal movies were analyzed using a custom MATLAB (version r2021b) code (found at https://github.com/janehumphrey/calcium). Segmentation of the cell membrane, segmentation of close contacts and calcium tracking from raw TIRF videos were analyzed using a custom python code (found at https://github.com/mkoerbel/contactanalysis_2D) using the packages NumPy, scikit-image, scipy, and pandas, as well as matplotlib and seaborn for visualization. TIRF images showing enrichment or exclusion of proteins from close contacts were analysed using a custom python code (accessory code found at https://github.com/mkoerbel/contactanalysis_2D). Fluorescence correlation spectroscopy data was analyzed using PyCorrFit (version 1.1.7; https://github.com/FCS-analysis/PyCorrFit). Flow cytometry was analyzed using FlowJo version 10.8.1. Statistical analysis was performed using Python statistical tool packages scipy and scikit-posthocs, or GraphPad Prism 9. Dose-response were generated using GraphPad Prism 9. Figure preparation was performed using Illustrator 2022.

For manuscripts utilizing custom algorithms or software that are central to the research but not yet described in published literature, software must be made available to editors and reviewers. We strongly encourage code deposition in a community repository (e.g. GitHub). See the Nature Portfolio guidelines for submitting code & software for further information.

Data

Policy information about availability of data

All manuscripts must include a data availability statement. This statement should provide the following information, where applicable:

- Accession codes, unique identifiers, or web links for publicly available datasets
- A description of any restrictions on data availability
- For clinical datasets or third party data, please ensure that the statement adheres to our policy

Most of the raw TIRF imaging data can be accessed at 10.5281/zenodo.7509910. Sample data for cell and calcium tracking using the confocal microscope can be accessed at 10.5281/zenodo.7510290. All additional datasets referred to in the current study are available from the corresponding authors on request. Source data are provided in the Source Data file.

Human research participants

Policy information about studies involving human research participants and Sex and Gender in Research.

Reporting on sex and gender	Sex and gender consideration were not required owing to the use of the Jurkat-derived cells lines to perform experiments. Primary cells were obtained from blood cones that do not provide information relating to the donor.
Population characteristics	*Describe the covariate-relevant population characteristics of the human research participants (e.g. age, genotypic information, past and current diagnosis and treatment categories). If you filled out the behavioural & social sciences study design questions and have nothing to add here, write "See above."*
Recruitment	*Describe how participants were recruited. Outline any potential self-selection bias or other biases that may be present and how these are likely to impact results.*
Ethics oversight	*Identify the organization(s) that approved the study protocol.*

Note that full information on the approval of the study protocol must also be provided in the manuscript.

Field-specific reporting

Please select the one below that is the best fit for your research. If you are not sure, read the appropriate sections before making your selection.

☒ Life sciences ☐ Behavioural & social sciences ☐ Ecological, evolutionary & environmental sciences

For a reference copy of the document with all sections, see nature.com/documents/nr-reporting-summary-flat.pdf

Life sciences study design

All studies must disclose on these points even when the disclosure is negative.

Sample size	No statistical calculations were used to predetermine sample sizes. We used large sample sizes for the field for confocal imaging (100s of cells per movie for calcium analysis and 10s-100s of cells for single confocal images) and for TIRFM (typically 10s of cells) to ensure robust statistics.
Data exclusions	No data were excluded from the analysis, except for cells that were on the edge of fields of view.
Replication	Experiments were typically replicated independently on at least three different days. All attempts at replication were successful. Sample sizes (number of cells, number of contacts, or number of field of views) and number of biologically independent experiments (or SLBs) are indicated in the corresponding figure legend.
Randomization	Cells were not randomly allocated into experiment groups as this was not required. To help control covariates, experimental repeats comparing SLB compositions, or comparing across different cell lines, were typically performed on the same day. When comparing across SLB compositions, we either performed a complete set of repeats on the same day (e.g., when comparing SLB2s with/without CD58 or ICAM-1) so that the state of the cells used was comparable, or when required to image across days (e.g., with an agonist pMHC titration on a given SLB composition), we first ensured the cells used were 'healthy' by ensuring most cells exhibited a calcium signal in response to OKT3-coated glass wells (~80% responding cells) such that results were comparable. The order in which SLB compositions or cell comparisons were performed was also altered for repeats. When comparing across cells, SLBs were prepared via a 'master mix' to ensure comparisons were specific to the cell line differences, rather than altered SLBs (owing, for example, to pipetting error). In addition, cells imaged during live confocal and TIRF imaging were randomly sampled as we could not control which cells landed in the field of view. Images of cells after live imaging were chosen at random and all cells within the field of view (except those on the edge) were used for analysis.
Blinding	Image acquisition from different cell lines or with different conditions were not blinded but relied on unbiased data collection from random regions in the wells or coverslip. Furthermore, all cells in a field of view (excep those on the edge of a field of view) were analyzed. Data analysis was not blinded and not required as imaging data was analyzed using custom codes for which the same parameters were applied across experimental groups in a given experiment i.e., it was an automated process.

Behavioural & social sciences study design

All studies must disclose on these points even when the disclosure is negative.

Study description	*Briefly describe the study type including whether data are quantitative, qualitative, or mixed-methods (e.g. qualitative cross-sectional, quantitative experimental, mixed-methods case study).*
Research sample	*State the research sample (e.g. Harvard university undergraduates, villagers in rural India) and provide relevant demographic information (e.g. age, sex) and indicate whether the sample is representative. Provide a rationale for the study sample chosen. For studies involving existing datasets, please describe the dataset and source.*
Sampling strategy	*Describe the sampling procedure (e.g. random, snowball, stratified, convenience). Describe the statistical methods that were used to predetermine sample size OR if no sample-size calculation was performed, describe how sample sizes were chosen and provide a rationale for why these sample sizes are sufficient. For qualitative data, please indicate whether data saturation was considered, and what criteria were used to decide that no further sampling was needed.*
Data collection	*Provide details about the data collection procedure, including the instruments or devices used to record the data (e.g. pen and paper, computer, eye tracker, video or audio equipment) whether anyone was present besides the participant(s) and the researcher, and whether the researcher was blind to experimental condition and/or the study hypothesis during data collection.*
Timing	*Indicate the start and stop dates of data collection. If there is a gap between collection periods, state the dates for each sample cohort.*
Data exclusions	*If no data were excluded from the analyses, state so OR if data were excluded, provide the exact number of exclusions and the rationale behind them, indicating whether exclusion criteria were pre-established.*
Non-participation	*State how many participants dropped out/declined participation and the reason(s) given OR provide response rate OR state that no participants dropped out/declined participation.*
Randomization	*If participants were not allocated into experimental groups, state so OR describe how participants were allocated to groups, and if allocation was not random, describe how covariates were controlled.*

Ecological, evolutionary & environmental sciences study design

All studies must disclose on these points even when the disclosure is negative.

Study description	*Briefly describe the study. For quantitative data include treatment factors and interactions, design structure (e.g. factorial, nested, hierarchical), nature and number of experimental units and replicates.*
Research sample	*Describe the research sample (e.g. a group of tagged Passer domesticus, all Stenocereus thurberi within Organ Pipe Cactus National Monument), and provide a rationale for the sample choice. When relevant, describe the organism taxa, source, sex, age range and any manipulations. State what population the sample is meant to represent when applicable. For studies involving existing datasets, describe the data and its source.*
Sampling strategy	*Note the sampling procedure. Describe the statistical methods that were used to predetermine sample size OR if no sample-size calculation was performed, describe how sample sizes were chosen and provide a rationale for why these sample sizes are sufficient.*
Data collection	*Describe the data collection procedure, including who recorded the data and how.*
Timing and spatial scale	*Indicate the start and stop dates of data collection, noting the frequency and periodicity of sampling and providing a rationale for these choices. If there is a gap between collection periods, state the dates for each sample cohort. Specify the spatial scale from which the data are taken*
Data exclusions	*If no data were excluded from the analyses, state so OR if data were excluded, describe the exclusions and the rationale behind them, indicating whether exclusion criteria were pre-established.*
Reproducibility	*Describe the measures taken to verify the reproducibility of experimental findings. For each experiment, note whether any attempts to repeat the experiment failed OR state that all attempts to repeat the experiment were successful.*
Randomization	*Describe how samples/organisms/participants were allocated into groups. If allocation was not random, describe how covariates were controlled. If this is not relevant to your study, explain why.*
Blinding	*Describe the extent of blinding used during data acquisition and analysis. If blinding was not possible, describe why OR explain why blinding was not relevant to your study.*

Did the study involve field work? ☐ Yes ☐ No

Field work, collection and transport

Field conditions	*Describe the study conditions for field work, providing relevant parameters (e.g. temperature, rainfall).*
Location	*State the location of the sampling or experiment, providing relevant parameters (e.g. latitude and longitude, elevation, water depth).*
Access & import/export	*Describe the efforts you have made to access habitats and to collect and import/export your samples in a responsible manner and in compliance with local, national and international laws, noting any permits that were obtained (give the name of the issuing authority, the date of issue, and any identifying information).*
Disturbance	*Describe any disturbance caused by the study and how it was minimized.*

Reporting for specific materials, systems and methods

We require information from authors about some types of materials, experimental systems and methods used in many studies. Here, indicate whether each material, system or method listed is relevant to your study. If you are not sure if a list item applies to your research, read the appropriate section before selecting a response.

Materials & experimental systems

n/a	Involved in the study	
☐	☒	Antibodies
☐	☒	Eukaryotic cell lines
☒	☐	Palaeontology and archaeology
☒	☐	Animals and other organisms
☒	☐	Clinical data
☒	☐	Dual use research of concern

Methods

n/a	Involved in the study	
☒	☐	ChIP-seq
☐	☒	Flow cytometry
☒	☐	MRI-based neuroimaging

Antibodies

Antibodies used	Anti-CD2-PE (BioLegend, Cat# 300208, 1:100), Anti-CD3-PE (BioLegend, Cat# 300408, 1:100), Anti-CD4-PE (BioLegend, Cat# 300508), Anti-CD8α-PE (BioLegend, Cat# 344706, 1:100), Anti-CD11a-PE (BioLegend, Cat# 350606, 1:100), Anti-CD45-PE (BioLegend, Cat# 304008, 1:100), Anti-HLA-A2-PE (BioLegend, Cat# 343306, 1:100), Anti-B2M-PE (BioLegend, Cat# 316306, 1:100), Anti-CD54-PE (BioLegend, Cat# 353105, 1:100), Anti-CD58-PE (BioLegend, Cat# 330905, 1:100), Anti-CD43-PE (BioLegend, Cat# 343203, 1:100), Anti-CD83-FITC (BioLegend, Cat# 305306, 1:100), Anti-CD1a-Pacific Blue (BioLegend, Cat# 300124, 1:100), Anti-CD14-PerCP/Cyanine 5.5 (BioLegend, Cat# 367110, 1:100), Anti-CD11c-AF647 (BioLegend, Cat# 301620, 1:100), Anti-HLA-DR-APC-Cy7 (BioLegend, Cat# 307618, 1:100), IgG1 Isotype-PE (MOPC-21; BioLegend, Cat# 400114, dilution matched to highest PE-labelled antibody concentration in staining experiment), Anti-CD62L (BioLegend, Cat #304802), HRP-conjugated primary anti-6xhis (Abcam, Ab1187), Anti-CD3 antibody (OKT3; Antibody purification service at Human Immunology Unit, WIMM, Oxford), Anti-CD45 antibody (Gap8.3; Antibody purification service at Human Immunology Unit, WIMM, Oxford), and Anti-CD3 Fab (UCHT-1; UCHT-1 hybridoma (generous gift from Dr. Neil Barclay, Sir William Dunn School of Pathology, University of Oxford).
Validation	Validation provided by supplier e.g., antibodies from BioLegend have been widely used and their use is cited on the BioLegend website. The specificity of the HRP-conjugated anti-6xhis can be found on the Abcam website. The specificity of the Anti-CD3 antibodies was validated by comparing TCR/CD3+ and TCR/CD3- Jurkat-derived cell lines.

Eukaryotic cell lines

Policy information about cell lines and Sex and Gender in Research

Cell line source(s)	Jurkat E6-1 (ATCC), HEK 293T (ATCC), primary CD8+ T cells were isolated from blood leukocyte cones (anonymous donors), and moDCs were generated from monocytes isolated from blood leukocyte cones (anonymous donors).
Authentication	The parental Jurkat E6-1.and HEK 293T cells were authenticated by the supplier (ATCC). Primary CD8+ and moDCs were authenticated by staining for key surface markers (CD3+CD8+ for primary CD8+ T cells, and CD14-CD1a+CD83+ for mature moDCs).
Mycoplasma contamination	Cell lines were routinely tested and found negative for mycoplasma contamination.
Commonly misidentified lines (See ICLAC register)	No commonly misidentified cell lines were used.

Flow Cytometry

Plots

Confirm that:

- [x] The axis labels state the marker and fluorochrome used (e.g. CD4-FITC).
- [x] The axis scales are clearly visible. Include numbers along axes only for bottom left plot of group (a 'group' is an analysis of identical markers).
- [ ] All plots are contour plots with outliers or pseudocolor plots.
- [ ] A numerical value for number of cells or percentage (with statistics) is provided.

Methodology

Sample preparation	Blood leukocyte cones purchased from the NHS Blood and Transplantation service at the John Radcliffe Hospital. Monocyte-derived dendritic cells (moDCs) were produced by isolating human monocytes from PBMCs using a Ficoll gradient and CD14+ CD16- magnetic bead isolation kit (Miltenyi). CD8+ T cells were isolated by Ficoll-Paque density gradient centrifugation followed by the CD8+ T Cell Isolation Kit (Miltenyi).
Instrument	Attune NXT (A24858)
Software	FlowJo (v10.8.1)
Cell population abundance	Any sorted cells (for example the J8-CD2KO cells, which have their CD2 ablated by CRISPR) were only used if they were >99% abundant. This was determined by staining with fluorescently-tagged antibodies and analyzing cells by flow cytometry.
Gating strategy	Jurkat-derived cell lines and primary T cells were first identified based on FSC-A/SSC-A, followed by FSC-A/FSC-H to exclude doublets. In the case of moDCs, cells were first sorted on live/dead stain followed by the above gating strategy for isolating single cells. The gated cells were then used to produce the flow histograms seen in the figures. Positive cells are defined by any signal above an isotype control/unstained cells.

- [x] Tick this box to confirm that a figure exemplifying the gating strategy is provided in the Supplementary Information.
